# Smartphone region-wise image indoor localization using deep learning for indoor tourist attraction

**DOI:** 10.1371/journal.pone.0307569

**Published:** 2024-09-09

**Authors:** Gabriel Toshio Hirokawa Higa, Rodrigo Stuqui Monzani, Jorge Fernando da Silva Cecatto, Maria Fernanda Balestieri Mariano de Souza, Vanessa Aparecida de Moraes Weber, Hemerson Pistori, Edson Takashi Matsubara

**Affiliations:** 1 Dom Bosco Catholic University, Campo Grande, MS, Brazil; 2 Federal University of Mato Grosso do Sul, Campo Grande, MS, Brazil; 3 Pantanal Biopark, Campo Grande, MS, Brazil; 4 State University of Mato Grosso do Sul, Campo Grande, MS, Brazil; 5 Kerow Precision Solutions, Campo Grande, MS, Brazil; Huazhong Agricultural University, CHINA

## Abstract

Smart indoor tourist attractions, such as smart museums and aquariums, require a significant investment in indoor localization devices. The use of Global Positioning Systems on smartphones is unsuitable for scenarios where dense materials such as concrete and metal blocks weaken GPS signals, which is most often the case in indoor tourist attractions. With the help of deep learning, indoor localization can be done region by region using smartphone images. This approach requires no investment in infrastructure and reduces the cost and time needed to turn museums and aquariums into smart museums or smart aquariums. In this paper, we propose using deep learning algorithms to classify locations based on smartphone camera images for indoor tourist attractions. We evaluate our proposal in a real-world scenario in Brazil. We extensively collect images from ten different smartphones to classify biome-themed fish tanks in the Pantanal Biopark, creating a new dataset of 3654 images. We tested seven state-of-the-art neural networks, three of them based on transformers. On average, we achieved a precision of about 90% and a recall and f-score of about 89%. The results show that the proposal is suitable for most indoor tourist attractions.

## 1 Introduction

The Pantanal Biopark (officially known in Portuguese as Bioparque Pantanal) is a state public aquarium in Campo Grande (20° 45’ 60.189” S latitude and −54° 58’ 07.123” W longitude), Mato Grosso do Sul, Brazil. It is the largest freshwater aquarium in the world, with 19 thousand m^2^ of built area and more than 5 million liters of water in 93 tanks, 70 of which are dedicated to research and conservation. More than 359 animal species from the Pantanal and other regions are housed in thematic tanks, offering visitors the opportunity to learn about the main Brazilian ecosystems, including chimney springs, floodplains and trails.

In addition to its touristic purpose, the Biopark is also designed as an educational facility where new tools are developed and made available to visitors. State-of-the-art technologies, particularly from the field of artificial intelligence (AI), are integrated into the visitor experience. One of these tools is a smartphone app that can act as a personal smart guide during the visit. The visitor’s location is a key feature that enables interaction with scenes and animals. Precise localization in indoor areas of the Biopark is a challenging task.

The architectural design of most indoor tourist attractions is usually made of metal structures and concrete. Conventional GPS methods are inaccurate in such locations as the structures block the GPS signal. Various technologies are available to solve indoor positioning systems in these cases. Best-known indoor positioning technology uses wireless data from relays and beacons, which requires the installation of expensive hardware infrastructure. Our proposal is a cost-effective solution that does not require hardware installation. The region-wise localization is achieved through image classification. The main idea is to combine the image classification of the fish tank with the prior knowledge of the arrangement of the tanks in the park, which enables a simple but effective indoor localization system.

The main contributions of this paper are:

- A novel image dataset with 3654 images from 24 different fish tanks (locations), 23 indoor locations, and one outdoor location of Biopark Pantanal;
- An experimental evaluation of seven deep learning algorithms, where we focused on the proposal’s applicability by selecting small and medium-size models that anyone with a standard GPU can train. Moreover, the small models can fit on any standard mobile phone device; and
- A discussion on how an indoor localization system based on the new dataset shall work.

## 2 Related works

**Fig 1:**
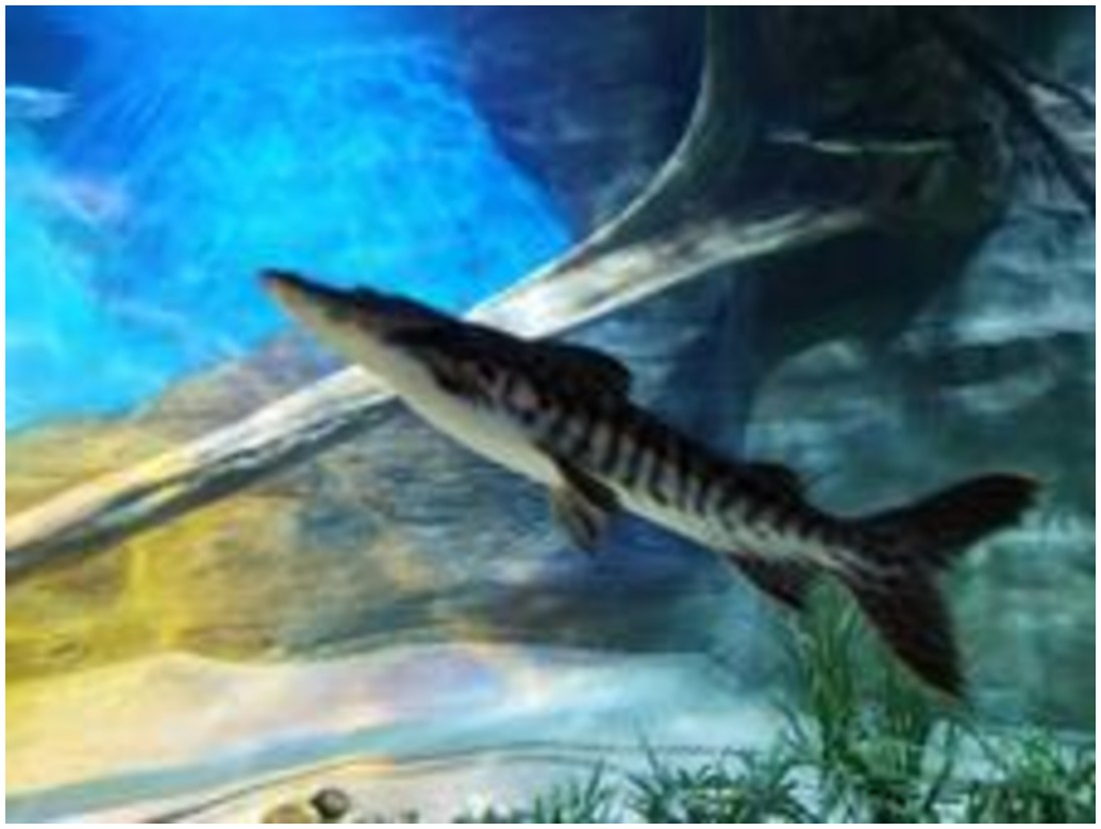
A fish in the *Banhados* tank.

The goal of the learned model in this paper is to consider as an input an image and the output is the location (fish tank name), which at Biopark Pantanal refers to the biomes that the tanks purport to mimic. The complex backgrounds and the fish species are the central features of the classification. It is also worth noting that images taken underwater through a glass panel, as is the case with images in an aquarium, have their peculiarities, such as light distortion due to the light coming through the water surface (see e.g. Fig 1). Work related to this study therefore includes aquarium classification, fish classification and indoor localization using deep learning.

Ubina *et al.* [1] addressed fish tank detection with deep learning techniques. In their work, the authors created a complex system using drones to automate different tasks (not only fish-related ones but also security surveillance tasks) in fish aquaculture sites. Their system did include the detection of aquaculture tanks using deep learning. Their problem was, however, clearly very different from the one addressed in this work, not only because of how the images were captured but also because the tanks were only classified amongst other kinds of objects (such as people and boats), and not amongst themselves, as tanks diversely themed according to different aquatic environments.

Most research on the application of deep learning in fish-related sciences up until now has focused on underwater images captured in marine environments. Zhao *et al.* [2] reviewed the application of machine learning techniques in the field of intelligent aquaculture, indicating that there are good results in the task of fish species classification (with an accuracy usually above 90%, frequently reaching 98%, and even going above 99% in at least one case reported in their work), but also that these results are highly dependent on the dataset and on the environmental conditions. The preferred datasets are the Fish4Knowledge dataset and versions of the LifeCLEF dataset. Many works address the complexity of the environments. Salman *et al.* [3], for example, addressed the issue of complex backgrounds (originating from complex environments) in underwater video processing by using a hybrid model consisting of gaussian mixture modeling and optical flow. Ju and Xue [4] also addressed the problem of complex backgrounds by proposing the Fish_AlexNet, a neural network based on the AlexNet, improved with an item-based soft attention mechanism. These works show that fish image classification is now a well-established field addressing its difficulties in their specificities.

Looking at indoor localization literature, Félix *et al.* [5] proposed in 2016 the use of deep learning on indoor localization. The authors evaluate the fingerprinting of the Wi-FI access point. Since 2016, deep learning models have evolved immensely, and now deep learning for mobile devices is a very feasible approach. Shao *et al.* [6] transformed Wi-Fi and magnetic field fingerprints into an image-like structure and processed it with a convolutional neural network. Their result shows an impressive under one-meter error distance using different smartphone orientations. Although the result is satisfying, they used Wi-Fi and magnetic fields as the primary input, which is not our case.

Liu *et al.* [7] proposed a system for indoor localization that uses both place and object recognition in images to map pictures into environments. Their primary input uses RGB images from the camera, where SURF, FLANN, and RANSAC preprocess the images to identify keyframes. The proposal submits the keyframes to a Deep Learning Object detection and performs the localization. Although the results are promising, their proposal requires server-client architecture to process all images due to its computational demands.

Bai *et al.* [8] presents a survey on deep learning image-based approach. Most works at that time do not implement deep learning end-to-end approaches but the combination of traditional computer vision methods (ex. SIFT, SURF, BOVW) with deep learning. Another interesting observation in this review is that most papers perform a multi-fusion of an image, WIFI fingerprinting, gyroscope, and accelerometer data.

The problem of classifying tanks seems to be an open question. Few steps have been taken lately toward its solution, and there are good reasons to speed up. Being in a different context, Sergi *et al.* [9] proposed a system that is related to ours in its spirit: enhancing educational tourism and learning experiences with deep learning. In their case, the proposed system used image-matching deep learning techniques to enhance the fruition of cultural tourism. The system can display information on cultural points of interest by matching user input into reference images stored in the system. In the case of this work, the techniques use image classification. As the authors say, it is possible to enhance the experience of learning about culture with artificial intelligence tools. The same can be said about biomes and aquatic fauna: it is possible to enhance the experience of learning about them with AI. Of course, the correct identification of biome-themed fish tanks is necessary. Therefore, in this step, we contribute by presenting an artificial neural network to classify biome-themed fish tank images.

## 3 Proposal

The proposal follows the standard pipeline of a fingerprint localization problem: it starts with image acquisition, followed by deep learning image classification to identify the fish tank, and finally the matching between the fish tank name and the localization. When accessing an indoor localization system based on smartphone cameras, a user captures an image of the tank and receives the localization as response. For example, for Fig 1, the system should inform the user that they are looking at the Wetlands-themed tank. The system can also return additional information and trivia about the tank and the species. Although this approach has been used in many different applications, our study is, to our knowledge, the first to evaluate this approach in a fish tank aquarium where specific indoor lighting and environmental conditions are present. We believe that this approach can be transferred to other similar indoor tourist attractions.

This paper considers two ways of processing input data: online and offline. For online processing, the possibilities range from cloud computing to local wireless access to a dedicated server in situ, where both allow the use of server GPUs and larger networks. For offline processing, it is possible to embed a smaller model in the smartphone application and run it on the user’s smartphone. There are many factors to consider with these options, particularly in the context of this work, such as the performance of smaller networks that can run in smartphones versus more extensive neural networks that require more computing power and may perform better. Therefore, we evaluate larger and smaller deep learning architectures to give the reader a broader understanding of the proposal’s applicability in both scenarios.

Using smaller deep learning models, it is possible to identify a tank based on a picture taken with a smartphone. According to our experimental evaluation, networks with fewer parameters also provide acceptable results for this task. Depending on the desired user experience trade-offs (response time and accuracy), the developers can choose between an online or offline approach.

## 4 Materials and methods

### 4.1 Dataset

**Fig 2:**
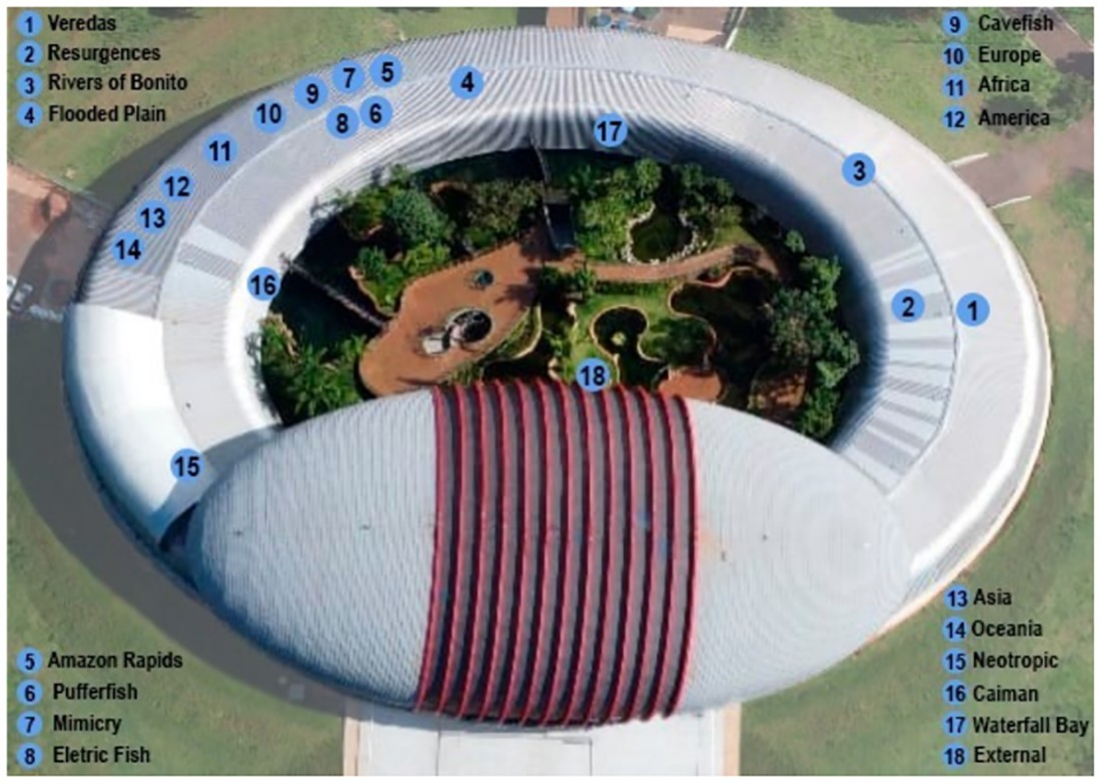
A 2D view of Pantanal Biopark, with the approximate location of each tank (Source: Authors).

The dataset classes are based on the Pantanal Biopark tank division. The tanks represented in the dataset are: Africa, America, Asia, Waterfall Bay, Pufferfish, Wetlands, Amazon Rapids, Europe, Creeks, Caiman, Australian Lake, Mimicry, Neotropic, Oceania, Electric Fish, Flooded Plain, Flooded Plain (Flooding), Resurgence, Rivers of Bonito, Rivers of the Pantanal, Veredas and External. Fig 2 shows the map of the fish tanks used in this proposal.

**Fig 3:**
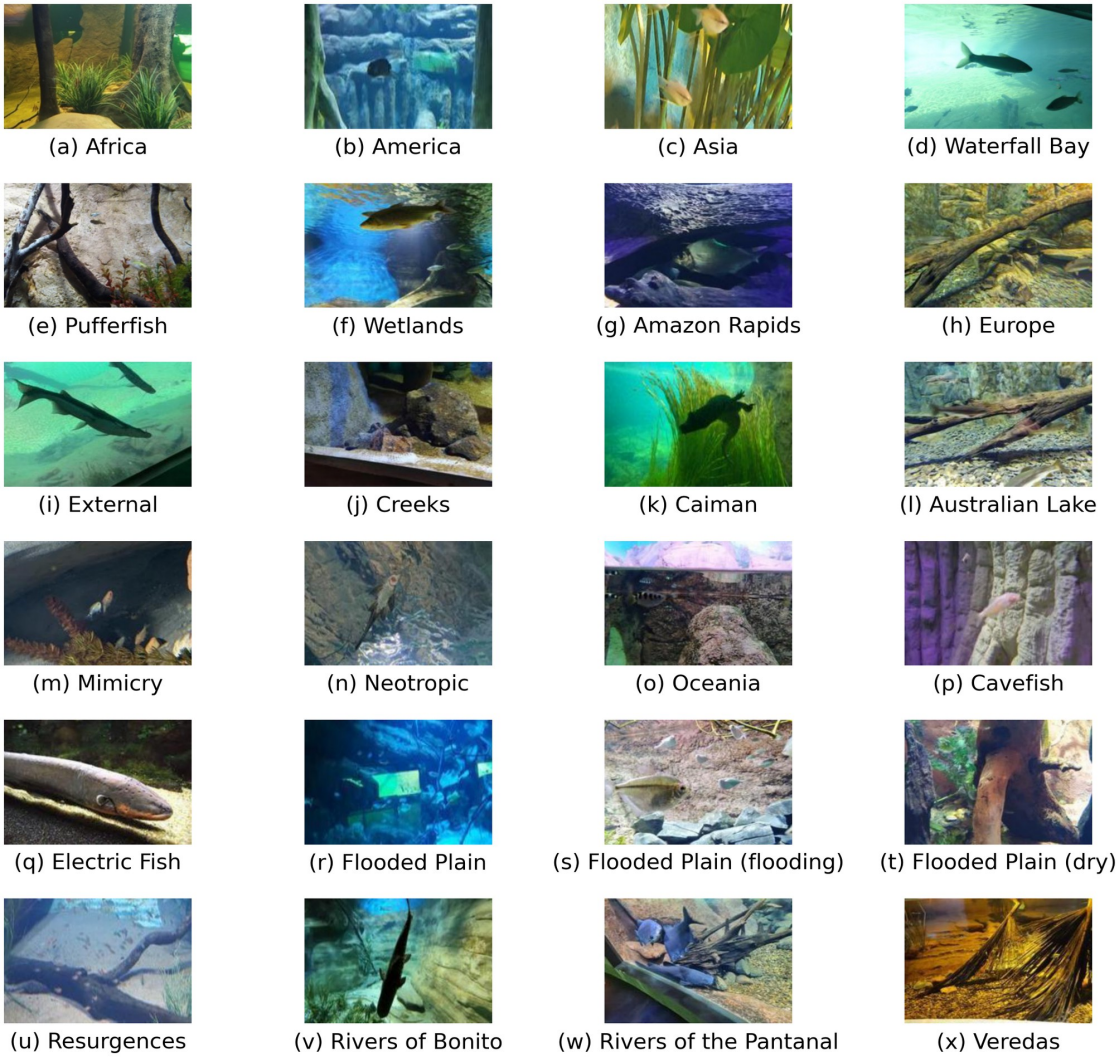
One image sample from each tank used in the experiment.

The images from the tanks were taken on May 21st, 2022, at 2 pm, by a group of 9 students from the Federal University of Mato Grosso do Sul (UFMS) in a tour guided by Prof. Edson Takashi Matsubara, who also contributed to the collection. Access to the location was granted by the chief executive of the Biopark, who is also a coauthor of this paper. The students were instructed to use their smartphones to collect as many images as possible with different angles and light conditions. In addition, it was also requested that there be a balance between the number of images recorded in each tank to avoid imbalance between classes. Table 1 shows the smartphone models, the resolutions of their cameras, and the number of images taken using each one, both in total and percentage. From the 31 tanks available, only 24 were used in this work, as the tanks with fewer than 50 images were removed because the deep learning techniques required more images to learn. In total, there are 3654 images of 24 tanks. Table 2 shows the number of images of each tank. In Fig 3, it is possible to see one sample image from each tank.

**Table 1:** Description of each smartphone used to build the dataset, including the number and percentage of images.

Model	Specification	# Images	Percentage
Apple iPhone XR	12 Mp	264	7.22%
Samsung S20+	12 Mp/64 Mp/12 Mp	316	8.65%
Realme 7 Pro	64 Mp/8 Mp/2 Mp/2 Mp	181	4.95%
Xiaomi Pocophone F1	12 Mp/5 Mp	109	2.98%
Redmi Note 8	48 Mp/8 Mp/2 Mp/2 Mp	148	4.05%
Samsung A30	16 Mp/5 Mp	316	8.65%
Samsung S7	12 Mp	627	17.16%
Samsung S22	50 Mp/12 Mp/10 Mp	481	13.16%
Xiaomi Mi 8 Lite	12 Mp/5 Mp	829	22.69%
Samsung M51	64 Mp/12 Mp/5 Mp/5 Mp	383	10.48%

**Table 2:** Number and percentage of images from each tank used in the experiment.

Tanks	# Images	Percentage
Africa	265	7.25%
America	196	5.36%
Asia	61	1.67%
Waterfall bay	170	4.65%
Pufferfish	110	3.01%
Wetlands	59	1.61%
Amazon rapids	78	2.13%
Europe	182	4.98%
External	212	5.80%
Creeks	116	3.17%
Caiman	85	2.33%
Australian lake	143	3.91%
Mimicry	116	3.17%
Neotropic	229	6.27%
Oceania	104	2.85%
Cavefish	81	2.22%
Electric fish	85	2.33%
Flooded plain	264	7.22%
Flooded plain (flooding)	233	6.38%
Flooded plain (dry)	95	2.60%
Resurgences	71	1.94%
Rivers of Bonito	413	11.30%
Rivers of Pantanal	172	4.71%
Paths	114	3.12%

Among the main species of each habitat, the following stand out:

- Africa: african cichlid, petricola catfish (*Synodontis petricola*) and featherfin chichlid (*Cyathopharynx furcifer*);
- America: triangle cichlid waroo (*Uaru amphiacanthoides*), porcupinefish (*Geophagus spp.*), true parrotfish (*Hoplarchus psittacus*),bandit cichlid (*Guianacara dacrya*), armored catfish, serverum (*Heros severus*), silver dollar (*Leporinus*) and pike cichlid (*Crenicichla spp.*);
- Asia: barb, clown loach (*Chromobotia macracanthus*), Hara jerdoni (Botia kubotai), Black shark minnow (Epalzeorhynchos bicolor), Three-striped corydoras (Trichopodus leerii), Aurelius Barb (Dawkinsia arulius), Golden barb (Barbodes semifasciolatus) and Yo-yo loach (Botia lohachata);
- Waterfall Bay: piraputanga (*Brycon hilarii*), armored catfish, Pantanal catfish (*Pimelodus pantaneiro*), swamp eel (*Synbranchus spp.*), schizodon (*Schizodon borellii*), silver dollar (*Leporinus friderici*), silver prochilod (*Mylossoma duriventre*), large-headed leporinus (*Megaleporinus macrocephalus*), and tortoise;
- Pufferfish: Amazon pufferfish (*Colomesus asellus*), greenbottle pufferfish (*Auriglobus nefastus*);
- Wetlands: piraputanga (*Brycon hilarii*), curimata-serrasalmid (*Prochilodus lineatus*), thicklip cichlid (*Cichasoma dimerus*), spotted pike cichlid (*Crenicichla lepidota*) and African butterfly fish (*Markiana nigripinnis*);
- Amazon Rapids: zebra pleco (*Hypancistrus zebra*), Hypancistrus catfish (*Hypancistrus sp.*), golden nugget pleco (*Baryancistrus xanthellus*), Baryancistrus catfish, cactus plecos (*Pseudacanthicus spp.*), sailfin plecos (*Leporacanthicus spp.*), flagtail prochilod (*Acnodon Normani*) and bloodfin tetra (*Moenkhausia heikoi*);
- Europe: rainbow trout (*Oncorhynchus mykiss*);
- Creeks: Panda corydoras (*Corydoras panda*), Neon tetra (*Paracheirodon innesi*), Green neon tetra (*Paracheirodon simulans*), Glowlight tetra (*Hemigrammus erythrozonus*) Nego d’água (*Hyphessobrycon negodagua*), Diamond tetra (*Moenkhausia pittieri*), Tetra Peugeot (*Hyphessobrycon Peugeot*), Lemon tetra (*Hyphessobrycon pulchripinnis*) Penguin tetra (*Thayeria boehlkei*), Costae tetra (*Moenkhausia costae*), Rummy-nose tetra (*Hemigrammus bleheri*), Cardinal tetra (*Paracheirodon axelrodi*), Spotted headstander (*Chilodus punctatus*), Cruzeiro do Sul (*Hemiodus gracilis*), Altum angelfish (*Pterophyllum altum*) and Discus fish (*Symphysodon discus*);
- Caiman: broad-snouted caiman (*Caiman Latirostris*), black belt cichlid (*Aequidens plagiozonatus*), flag acara (*Bujurquina vittata*), thicklip cichlid (*Cichlasoma dimerus*), black tetra (*Moenkhausia dichroura*) and silver dollar fish (*Tetragonopterus argenteus*);
- Australian Lake: Rainbowfish;
- Mimicry: Dwarf corydoras (*Corydoras hastatus*), Lips (*Hemigrammus mahnerti*), Krieg’s tetra (*Serrapinnus kriegi*), Serpae tetra (*Hyphessobrycon eques*), Black Phantom tetra (*Hyphessobrycon megalopterus*), Lips (*Hyphessobrycn elachys*) and Armored catfish;
- Neotropic: Brazilian dourado (*Salminus brasiliensis*), stingray, pintado (*Pseudoplatystoma corruscans*), Tambaqui (*Colossoma macropomum*), thorny catfish (*Oxydoras kneri*), Astyanax (*Astyanax lacustres*), spine-bellied jupiaba (*Jupiaba acanthogaster*), Pirarucu (*Arapaima gigas*), disk tetra (*Myleus schomburkii*), armored catfish, silver dollar, red head tapajos (*Geophagus spp.*), silver arowana (*Osteoglossum bicirrhosum*), redtail catfish (*Phractocephalus hemeliopterus*) and pike cichlid (*Crenicichla spp.*);
- Oceania: archerfish (*Toxotes jacutrix*), archerfish (*Toxotes jaculatrix*), fingerfish (*Monodactylus sabae*) and silver moony (*Monodactylus argenteus*);
- Electric Fish: Black ghost knifefish (*Apteronotus spp.*);
- Flooded Plain: Marbled headstander (*Abramites hypselonotus*), Armored catfish, stingray, Canivete (*Leporinus striatus*), Catfish (*Auchenipterus osteomystax*), serrasalmid (*Myloplus levis*), Pejerrey (*Schizodon isognathus*), Black tetra (*Gymnocorymbus ternetzi*), Argentine humphead (*Gymnogeophagus balzanii*), Astyanax (*Astyanax alleni*), Sardinha (*Triportheus pantanensis*) and whiptail armored catfish (*Loricaria spp.*);
- Flooded Plain (Flooding): papudinho (*Poptella paraguayensis*), Astyanax (*Psalidodon marionae*), flag cichlid (*Mesonauta festivus*) and Redeye tetra (*Moenkhausia forestii*);
- Resurgences: jewel tetra (*Hyphessobrycon eques*), whiptail armored catfish, tetra (*Moenkhausia bonita*) and saguiru (*Toothless characin*);
- Rivers of Bonito: Piraputanga (*Brycon hilarii*), thicklip cichlid (*Cichlasoma dimerus*), Pike cichlid (*Crenicichla lepidota*), Armored catfish, stingray, ray-finned fish (*Prochilodus lineatus*), Astyanax (*Psalidodon marionae*, *Jupiaba acanthogaster*, *Hyphessobrycon luetkeni*, *Moenkhausia bonita*), Canivete (*Leporellus vittatus*, *Leporinus striatus*), Duro-duro (*Parodon nasus*), Headstander (*Megaleporinus obtusidens*), stingray and tiger shovelnose catfish (*Pseudoplatysto reticulatum*);
- Rivers of the Pantanal: thorny catfish (*Oxydoras kneri*), stingray (*Potamotrygon spp.*), Armored catfish, Gilded catfish (*Zungaru jahu*), Brazilian dourado (*Salminus brasiliensis*), serrasalmid (*Piaractus mesopotamicus*), pike characin (*Acestrorhynchus pantaneiro*) and Pterodoras granulosus (*Pterodoras granulosus*);
- Veredas: Armored catfish, Astyanax (*Astyanax sp.*), Tetra (*Hyphessobrycon langeani*), Angelfish (*Aequidens sp.*), Chum-chum (*Pimelodella spp.*) and Silver Dollar (*Leporinus sp.*);
- External: Armored catfish, Jurupensém (*Sorubim lima*), Oscar (*Astronotus crassipinnis*), serrasalmid (*Metynnis mola*), Sardinha (*Triportheus pantanensis*), headstanders (*Leporinus lacustris*) and Pike cichlid (*Crenicichla vittata*);

### 4.2 Neural networks

The task at hand is classifying tank images according to the thematic tanks. In the field of computer vision, the use of convolutional neural networks has achieved remarkable results. In the last few years, another similar but ultimately different kind of technology has become an object of interest in the field: vision transformers.

In 2017, the work of Vaswani *et al.* [10] in the field of natural language processing resulted in this new kind of neural network, the transformers, a smaller neural network that achieved state-of-the-art results by making use of an attention mechanism called *scaled dot-product attention* and much fewer convolutional layers. In 2020, Dosovitskiy *et al.* [11] proposed a sort of “pure” transformer for computer vision tasks, precisely the Vision Transformer (ViT). Recognizedly, their work was not the first in which the use of transformers was attempted in Computer Vision. However, as the authors claim, it was the first that showed that a transformer could be used in a “pure” state (*i.e.*, without mixing a transformer-based architecture with a convolutional approach) and achieve state-of-the-art results.

Vision transformers have become a hot topic in Computer Vision ever since. New transformer-based architectures and new transformer-with-convolution-based architectures have been proposed in the last few years. Given that this is how things stand, the proposed experiment shall compare the performance of the following neural networks: Resnet18, MaxViT, LamHaloBotNet, LambdaResnet. Furthermore, given the proposal described in Section 3, we also test and compare three relatively smaller networks: EfficientNet, DenseNet and MobileNet. All networks will be compared regarding their performance differences given their sizes, which will form the basis for deciding whether the networks should be processed online or on the users’ smartphones.

Below, we comment briefly on each one of the architectures evaluated. Also, Table 3 shows the number of parameters of each network. This refers to the total number of parameters in each neural network, which is one of the aspects that determine the computational requirements both for training and for using neural networks. In general, a smaller number of parameters means a faster and lighter neural network.

- Resnet18: Residual networks were proposed by He *et al.* [12] in 2015 as a way to deal with the fact that the increase in depth of a neural network eventually leads to a degradation in accuracy, when the network gets too deep. The main idea is to adjust the weights in a group of layers with reference to the input given to that group. This reference is accomplished by adding the input itself (as it originally is, or linearly transformed if so required by the dimensionality) to the output of the layers, which makes optimization easier. In this work, we use the PyTorch/Torchvision implementation.
- MaxViT: Multi-Axis Vision Transformers were proposed in 2022 by Tu *et al.* [13]. In fact, the authors proposed, first and foremost, a transformer module called multi-axis self-attention, and what is here called MaxViT is the neural network, also proposed by the authors, composed by blocks constituted by the module and convolutional layers. It is, therefore, a hybrid architecture, both transformer- and convolution-based. In short, the fundamental idea of the new block is to use block attention, attention over windows of the input map, to get local information, and grid attention, attention diffused across smaller pieces distributed across the input map, to get global spatial information. The purpose is to improve the model capacity of the transformer, while keeping computational costs low. In this work, we tested the MaxViT-T as implemented in PyTorch Image Models [14] (timm; baptized in implementation as maxvit_rmlp_tiny_rw_256), available here: https://github.com/rwightman/pytorch-image-models.
- LambdaResnet: Lambda layers, as well as LambdaResnets, were proposed in 2021 by Bello [15]. LambdaResnets are Resnets in which the bottleneck blocks were substituted by lambda layers. Lambda layers were proposed as a way to model contextual information out of the self-attention framework by transforming contexts into linear functions to be applied on the queries. By doing this, lambda layers work as an attention mechanism that requires less memory, and can improve performance on some tasks, specially when compared with transformer-based models. Both LambdaResnet and LamHaloBotNet are available here: https://github.com/rwightman/pytorch-image-models as well. Wightman [14], maintainer of the implementations, considers them “experimental variants” implemented in a “bring-your-own-blocks” fashion.
- LamHaloBotNet: HaloNets were proposed in 2021 by Vaswani *et al.* [16]. The authors explore the similarities between self-attention and convolution, and propose to apply self-attention by getting queries from blocks established on the input, and keys and values from a window created around the query block, including this block itself. Bottleneck transformers are a kind of architectural design whose importance was pointed out by Srinivas *et al.* [17]. In short, it consists in the possibility of replacing convolutions with the transformer’s multi-head self-attention in certain cases, such as that of the bottleneck block of ResNets.
- EfficientNet: the family of EfficientNet networks was proposed by Tan and Le [18] as the result of a research on how to efficiently scale deep neural networks up. In their work, the authors proposed a compound scaling method to scale network depth, width and resolution in a uniform way. To test their method, they proposed a baseline EfficientNet-B0, along with efficiently scaled up versions from B1 up to B7. In this work, we evaluate the B2 version.
- MobileNetV3: the MobileNetV3 networks were proposed by Howard *et al.* [19], and constitute the third generation of MobileNets. This family of networks was conceived within the ideal of optimizing neural networks for mobile devices. In this third generation, the authors used two techniques for automated network architecture search, platform-aware NAS and NetAdapt, in order to find a network that minimizes latency while keeping a certain level of accuracy. Furthermore, after applying these algorithms, the authors also introduced important changes in aspects that were not in the automated search space, such as the redesign of computationally expensive layers and the use of hard swish as non-linearity, which is less costly than the standard swish function, specially in embedded systems.
- DenseNet121: Densely Connected Convolutional Networks were introduced by Huang *et al.* [20]. In their work, the authors proposed a network design where the output feature maps of each layer are used as inputs to all subsequent layers, and all subsequent layers have as inputs the feature maps of all preceding layers, up until the input image. The objective was to improve the flow of information and gradient throughout the layers, by sharing the feature maps across the entire network. With this idea, the authors managed to achieve results that were close to the state-of-the-art with a smaller number of parameters. In this work, we used the Pytorch/Torchvision implementation.

**Table 3:** Number of parameters of each neural network evaluated in this work.

Architecture	# Parameters
Resnet18	11,689,512
MaxViT	29,148,896
LambdaResnet	10,988,688
LamHaloBotNet	22,569,824
EfficientNet	9,109,994
MobileNetV3	5,483,032
DenseNet121	7,978,856

As one can see in Table 3, MobileNetV3 is the smallest model we evaluated in this study, with less than 5.5 million parameters. While there are smaller neural networks than the MobileNetV3 we evaluated, which still has over 5 million parameters, this network was chosen by us for this evaluation because of its being widely used in contexts where computing power is limited, either as it is or as a basis for the development of a new architecture. In fact, since its introduction it has been evaluated regarding its usage in mobile devices, with a reported latency of down to 44 ms in a Pixel 3 smartphone for the large version. It has been further utilized in other works where efficiency is important, such as those by Yang and Han [21] and by Tian *et al.* [22]. In the first case, MobileNetV3 was modified and used as backbone for a YoloV3 detection model. In the second case, it was modified and the number of parameters was reduced to less than a million, with a reported inference time of 26.4 ms for one image.

### 4.3 Experimental setup

The selected network architectures’ weights were adjusted by the Adaptive Moment Estimation (Adam) optimizer. The combinations were tested using a stratified 10-fold cross-validation sampling strategy. In each fold, 20% of the training data was used for validation. The maximum possible number of epochs was 1000. However, validation loss values were monitored for early stopping, with a tolerance of 0.1 and 30 epochs of patience. The architectures were initialized with the available pre-trained weights (transfer learning) in all cases. The images were resized to (256, 256) since this is the required image size for all the neural network implementations utilized. The following data augmentation techniques were used: random horizontal and vertical flips, with a probability of 0.5; random 90-degree rotation; random crop; and random perspective alterations (these are implemented in Torchvision as RandomHorizontalFlip, RandomVerticalFlip, RandomRotation, RandomCrop, and RandomPerspective). The weights were optimized using a learning rate of 0.001. The training was performed in batches of 12 images.

The results were used to calculate statistics for three performance metrics: precision, recall, and f-score. A one-way ANOVA hypothesis test was used to compare the effects of architecture variation on each performance metric. The chosen significance level was 5%. The Scott-Knott clustering test followed each ANOVA to specify the statistically different architectures. The Scott-Knott test is an alternative to traditional multiple comparisons tests, such as TukeyHSD. It makes use of a clustering method, followed by a likelihood test in order to create groups with homogeneous means, thus allowing for the appreciation of statistically significant differences between means [23]. For further details, the reader is referred to the specialized literature, such as the paper by Scott and Knott [23]. Readers who are more implementation-oriented can also check out the paper by Jelihovschi *et al.* [24]. The ANOVA and the post-hoc Scott-Knott tests were conducted in the R programming language. For the ANOVA, the R Stats Package was used. The Scott-Knott clustering test was performed with the ScottKnott package [24].

Boxplots, confusion matrices and Receiver Operating Characteristic (ROC) curves were also produced and used in the analysis. In order to calculate the ROC curves for multiclass classification, both the strategies of micro-average and macro-average were used. In the first case, true positives, false positives, true negatives and false negatives were calculated for each class in a one-vs-rest strategy, and the ROC curves were generated after that. In the second case, one ROC curve was calculated for each class, also in a one-vs-rest strategy, and the resulting ROC curves were averaged. The usage of both averaging strategies allowed for a better evaluation of the ROC curves, as well as of the ROC-AUC, in a multiclass classification context. The difference in how both averages are calculated also helps us understand the effect of dataset imbalance on the ROC results.

## 5 Results and discussion

**Fig 4:**
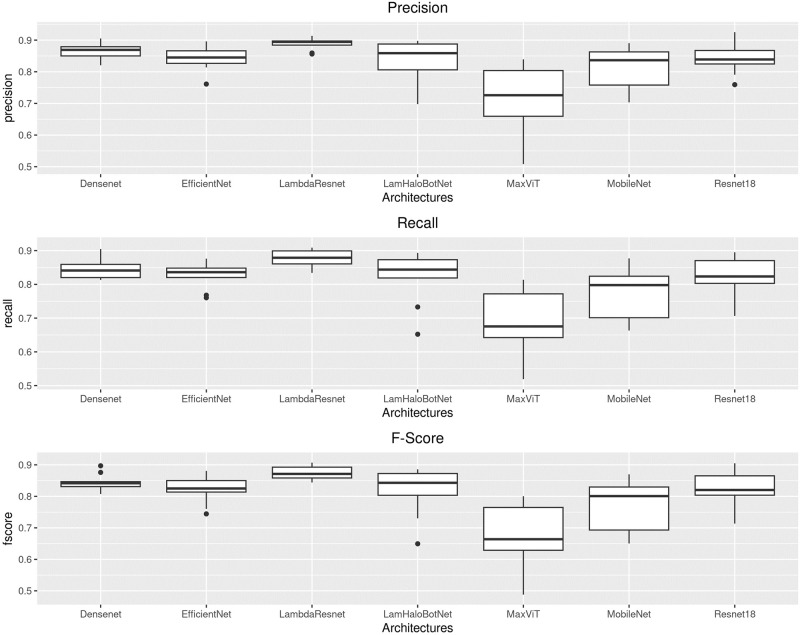
Boxplot for each architecture used in the experiment.

**Fig 5:**
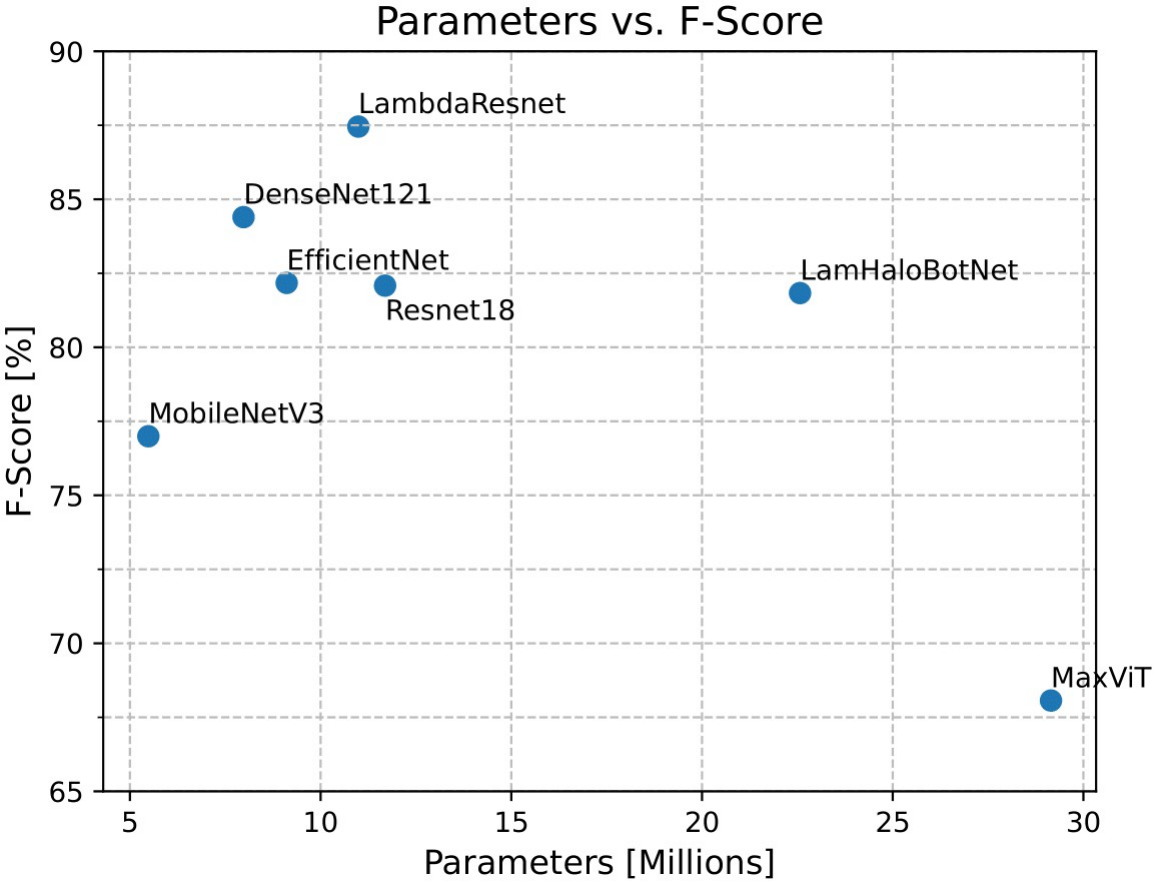
Number of parameters vs. f-score results for each architecture.

Fig 4 shows boxplots for precision, recall, and f-score for each architecture across ten folds. Table 4 shows the median, interquartile range (IQR), mean, and standard deviation (SD) for precision, recall, and f-score for each architecture. Fig 5 compares the number of parameters of each neural network with the f-score results achieved by them. Considering the convex hull of the graph, MobileNetV3, DenseNet121, and LambdaResnet are the best trade-off of f-score and number of parameters.

**Table 4:** Precision, recall and f-score statistics for each architecture. Scott-Knott clustering test results are presented as lowercase letters next to the mean values.

**Precision**				
Architecture	Median	IQR	Mean	SD
Resnet18	0.8388610	0.0427257	0.8431657 a	0.0488431
MaxViT	0.7258782	0.1445138	0.7181872 b	0.1013066
LambdaResnet	0.8943776	0.0129604	0.8891400 a	0.0190454
LamHaloBotNet	0.8587547	0.0815401	0.8371838 a	0.0646207
EfficientNet	0.8450530	0.0396771	0.8416580 a	0.0376566
MobileNetV3	0.8364602	0.1048045	0.8118989 a	0.0654814
DenseNet121	0.8692268	0.0290350	0.8665466 a	0.0268982
**Recall**				
Architecture	Median	IQR	Mean	SD
Resnet18	0.8234125	0.0676031	0.8222047 a	0.0594642
MaxViT	0.6753436	0.1295495	0.6924280 c	0.0916703
LambdaResnet	0.8787862	0.0385103	0.8775279 a	0.0253155
LamHaloBotNet	0.8438065	0.0541796	0.8224366 a	0.0750914
EfficientNet	0.8360617	0.0279304	0.8268298 a	0.0367740
MobileNetV3	0.7977565	0.1230585	0.7743310 b	0.0786110
DenseNet121	0.8410389	0.0391698	0.8431819 a	0.0286452
**F-score**				
Architecture	Median	IQR	Mean	SD
Resnet18	0.8201954	0.0615506	0.8208287 a	0.0614754
MaxViT	0.6638640	0.1360363	0.6806592 b	0.0962083
LambdaResnet	0.8712313	0.0343017	0.8745617 a	0.0212171
LamHaloBotNet	0.8429809	0.0691919	0.8183138 a	0.0762315
EfficientNet	0.8249585	0.0364336	0.8217447 a	0.0419911
MobileNetV3	0.8007643	0.1366063	0.7699452 a	0.0809824
DenseNet121	0.8415972	0.0158589	0.8439717 a	0.0258583

The ANOVA test resulted in highly significant results, with *p*-values of 4.9 ⋅ 10^−7^ for precision, 2.46 ⋅ 10^−7^ for recall and 1.31 ⋅ 10^−7^ for f-score. Also, in Table 4, the results of the Scott-Knott clustering test applied for each metric are presented next to the mean values. The architectures with the same letters were clustered and are not statistically different. Therefore, one can see that, for precision, since only MaxViT was in the ‘b’ group, there is statistical evidence that MaxViT, and only MaxViT, belongs to a group with inferior precision results. For recall, MaxViT is in the bottom group, ‘c’, but MobileNetV3 is in an intermediary group, ‘b’. Finally, for f-score, the situation is analogous to that of precision, with MaxViT alone in the ‘b’ group. Regarding the architectures in the ‘a’ group, there is no statistical evidence that their performance with respect to each metric differ, and a practical choice could be based on other criteria, such as the number of parameters and inference time.

As stated, there is evidence for asserting that MaxViT yielded a lower performance. After that, another result that deserves highlighting is the one achieved by the MobileNetV3. While the Scott-Knott test suggests that it is not as competitive regarding recall, when compared with the other architectures, there is no evidence that its performance is lower regarding precision and f-score. This, along with the fact that EfficientNet and DenseNet121 are also ‘a’ group architectures, suggests that smaller architectures can be viable for solving the problem. Therefore, further research could also investigate even smaller neural networks, as well as the possibility of reducing the size and computing requirements of the ones evaluated in this study.

**Fig 6:**
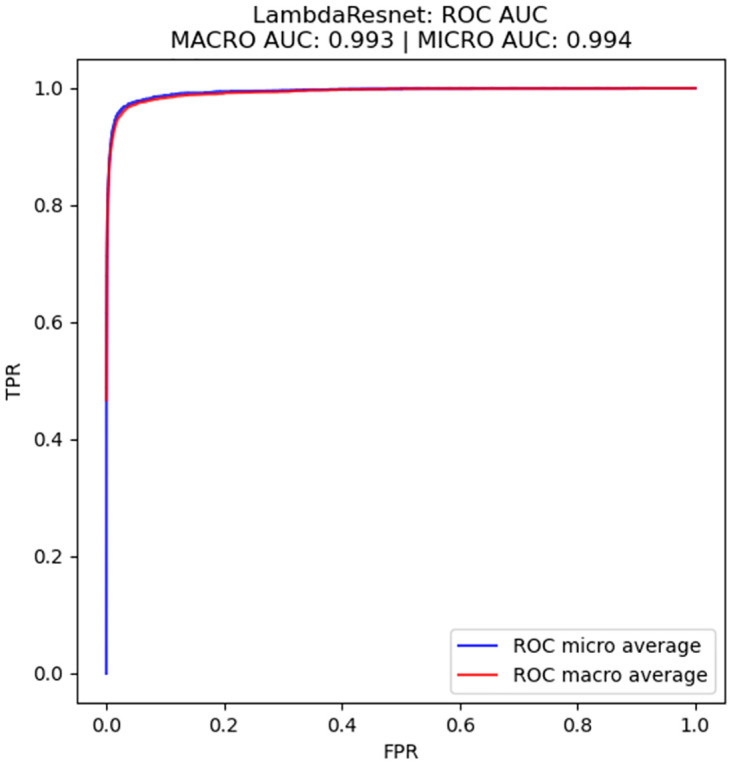
ROC curves calculated as micro and macro averages for the LambdaResnet architecture.

**Fig 7:**
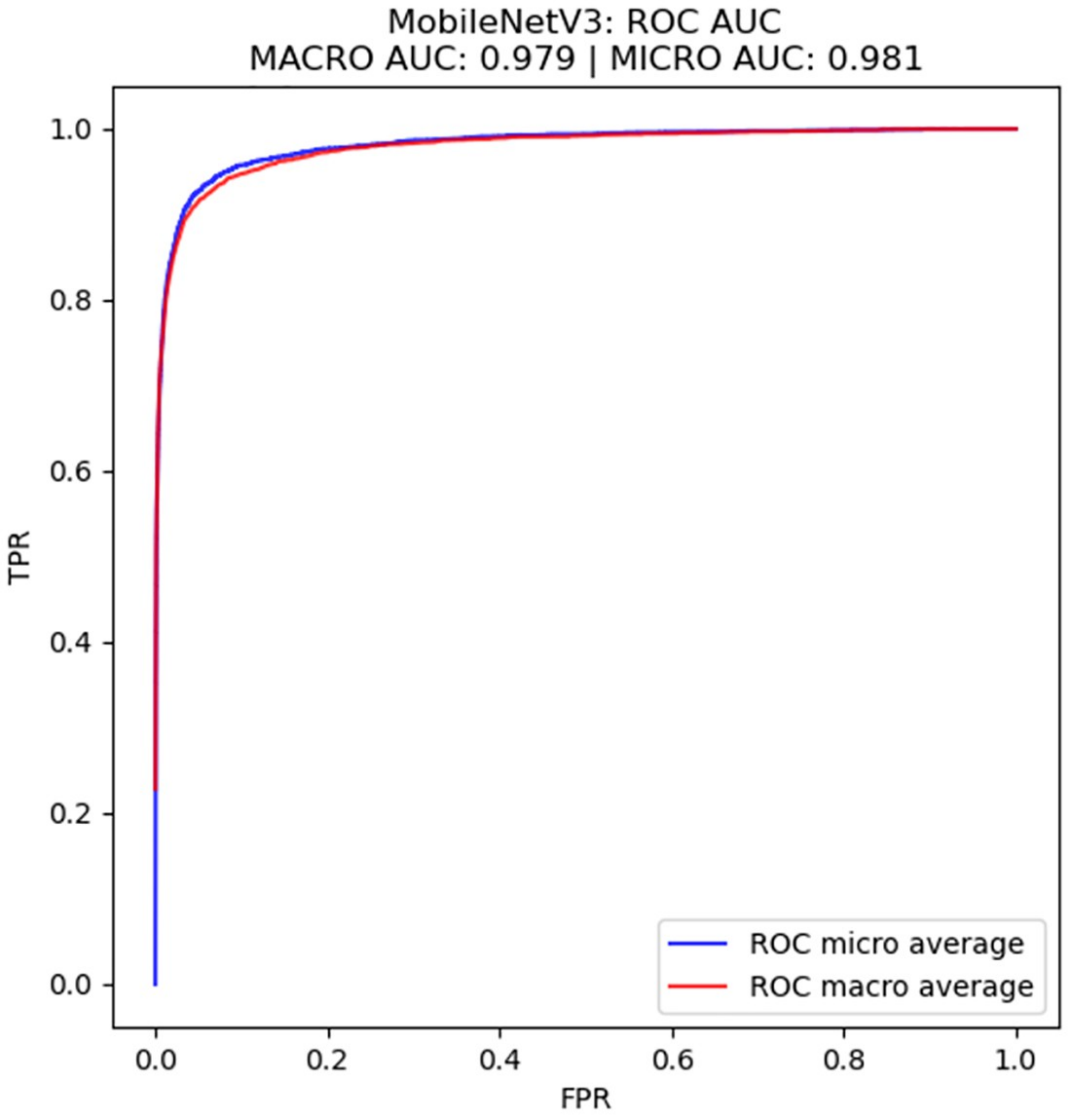
ROC curves calculated as micro and macro averages for the MobileNetV3 architecture.

Figs 6 and 7 show the Receiver Operating Characteristic (ROC) curves for LambdaResnet (11 million parameters), which achieved the highest average performance, as it is possible to see in Table 4, and for the MobileNetV3 architecture (5.4 million parameters), which is the smallest architecture evaluated in this work, as can be seen in Table 3. These curves were calculated with a one-vs-rest strategy, which means that the number of classes and the fact that they are imbalanced may have affected the results, although the fact that the micro- and macro-averaged ROC AUC were very similar, with differences of 0.001 to 0.002, is an indicative that this is not the case. Nonetheless, the ROC curves present an AUC higher than 0.98, indicating that the results of f-score can be even higher when using different classification thresholds. In both cases, we can see a steady increase in FPR, which allows us to set a classification threshold to remove false positives from the localization.

Regarding the proposed framework, one should notice that a downside of the approach used is that the neural networks would require retraining if the scenarios were to change, which is an important limitation. Fish tanks in the Biopark are unlikely to change, since they are fixed in their places and were carefully designed to represent certain biomes. Furthermore, one could notice that since the classification targets are the tanks themselves, a fitting model could be expected to ignore the foreground objects, the fishes, and focus on the background, which is the proper target. Therefore, one possible objection, that of new fish species being added to the tank leading to a full retraining, should be regarded as an additional difficulty of the problem with respect to the capability of the model, rather than a major impediment. New fish species shouldn’t impact model performance, although evaluating this would require a different experimental design, which is left for future research. On the other hand, the question of changing scenarios does remain a problem for other locations, even within the Biopark itself, where cultural exhibitions and other events can be realized. In those cases, the scenarios are likely to change. This problem was not addressed in the current work, and shall also be left for future research.

One possible solution for these problems is the integration between computer vision models and proper indoor localization techniques. Frameworks such as VITAL, proposed by Gufran *et al.* [25], DeepRSSI, proposed by Yoon *et al.* [26], as well as that proposed by Nguyen *et al.* [27]. In this case, computer vision models could be dedicated to some more specific tasks, such as fish detection and fish species classification. On the proposed framework, however, we focused on classifying fish tanks and performing localization therewith. Arguably, classifying fish tanks may also be useful, regardless of the possibility of indoor localization, if the goal is to enhance the educational experience, since some fish species live in different biomes, and since presenting information on biomes may also be important. Since some fish tanks are close to others, techniques such as fingerprint localization may not always be feasible to know what is the tank a person is interested in, even if their location is known with high accuracy. This may happen, for instance, when a person is interested in the Veredas tank, which is right in front of, and close to, the Resurgences tank, as one can see in Fig 2. That being said, further research will be necessary both in order to evaluate the performance of such indoor localization methods in the task at hand, and also in order to devise a specific way to combine fish vision and indoor localization, which is a promising way to solve the problem presented here.

Three last observations regarding the dataset: first, in Section 4, we said that the students who captured the images were instructed to keep the dataset balanced. However, this did not happen, as seen in Table 2. According to the students themselves, this happened because some fish were too small, and some students insisted on taking a picture of such fish. In order to do so, they took more pictures of tanks with such small fish than of others. Therefore, the imbalance in the dataset can be understood as a characteristic of the problem reflected in the dataset. Secondly, the natural behavior of the students also caused the dataset to have some sets of images that are too similar to each other, which may affect the results, but is also an aspect of the problem. Third, and finally, since different smartphone models were used to compose the dataset, as can be seen in Table 1, it is possible that the results are influenced not only by the natural conditions under which the data were collected, but also by the different specifications and configurations of the cameras that were used for data collection. It is not clear whether this is a major issue, but if it is proven to be relevant, a future research can evaluate this by separating the dataset according to the smartphone, since this information is available in the dataset.

## 6 Conclusion

This work was motivated by the possibility of improving the experience of learning about aquatic environments by visiting the Pantanal Biopark, the largest freshwater aquarium in the world. As reported, the task of using AI to improve visiting experience in cultural contexts is not without precedent, and in this work it was our intention to give an important step towards such a goal in the context of the Biopark. The proposal presented by us was to use deep learning models for image classification in an indoor localization strategy. More specifically, in this work we presented a new dataset composed by images of many of the tanks present in the Biopark. The task that comes with the dataset is to correctly identify the tanks from which the images were taken. We argued that, by knowing the location of each tank, and by correctly classifying the tanks, it is possible to perform indoor localization. The overarching assumption is that by integrating artificial intelligence into the system, the learning experience can be improved. By applying some state-of-the-art neural network architectures, we established some baseline results of around 88% for precision, recall and f-score. Although not bad, these results are still not ideal to build a system with the proposed objectives. As discussed, it is our opinion that there is room for improvement, both by testing other architectures and optimizers, and also by testing different hyperparameters for the same architectures and for the Adam optimizer. It is also possible that high performance, enough for a commercial system to be developed, will be achieved by integrating traditional indoor localization approaches with computer vision models. Indoor localization approaches alone may not be the ideal solution, since some of the objectives require not only localization, but also the determination of which tank the user is interested in, which may be performed by the proposed approach. In this case, using indoor localization methods could reduce the responsibilities of computer vision models and allow them to be specialized in fish vision. These questions are left open for future research.
